# Parental and Staff Experiences of Participation in the REPORT-BPD Feasibility Study: Insights from an Embedded Qualitative Research

**DOI:** 10.3390/healthcare13212694

**Published:** 2025-10-24

**Authors:** Wisam Muhsen, Ana Guillot-Lozano, Jos M. Latour

**Affiliations:** 1 Faculty of Health, University of Plymouth, Plymouth PL4 8AA, UK; ana.guillotlozano@students.plymouth.ac.uk (A.G.-L.); jos.latour@plymouth.ac.uk (J.M.L.); 2 Department of Nursing, Zhongshan Hospital, Fudan University, Shanghai 200031, China; 3 Curtin School of Nursing, Curtin University, Perth, WA 6102, Australia

**Keywords:** qualitative research, parental experiences, healthcare professional experiences, bronchopulmonary dysplasia, echocardiography, preterm infants, feasibility study, family-centred care

## Abstract

**Highlights:**

**What are the main findings?**

- Parents exhibited a clear understanding of the study’s aims and procedures and expressed confidence in its safety, whereas healthcare professionals displayed inconsistent awareness.
- Both parents and staff valued participation, highlighted emotional and logistical challenges in the neonatal unit environment, and offered practical suggestions to improve future neonatal research engagement.

**What are the implications of the main findings?**

- Sustained, multi-directional communication is essential to maintain staff engagement and ensure consistent understanding across all research stakeholders.
- Embedding family-centred, emotionally supportive strategies—such as structured reflective tools and timely feedback—can enhance ethical recruitment, retention, and data quality in future neonatal feasibility trials.

**Abstract:**

**Background/Objectives:** Family-centred care is key in neonatal practice, yet parents’ and staff’s research experiences are understudied. This study aims to explore their perspectives to improve inclusiveness, communication, and effectiveness in future neonatal research design and implementation. **Methods**: This embedded qualitative study, conducted within the Right vEntricular function applicability in a Prediction mOdel to identify pReterm infanTs with early BronchoPulmonary Dysplasia (REPORT-BPD) feasibility study, employed a qualitative descriptive design. The sample included 10 healthcare professionals, evenly split between medical and nursing backgrounds, and 10 parents, equally distributed between mothers and fathers of preterm infants enrolled in the REPORT-BPD study. Data were collected through audio-recorded semi-structured interviews, then transcribed into Word, and imported into NVivo 14 for thematic analysis by three researchers. **Results**: The following four main themes were developed from 11 sub-themes that were initially extracted: (1) Trust and Assurance in the Study, reflecting the overall trust between parents and staff, emphasising its perceived study’s safety and minimal impact on the infant. (2) Emotional and Psychological Considerations, highlighting the emotional landscape of parents, including their anxieties, stressors, and support systems that help ease their concerns. (3) Communication and Engagement, underscoring the importance of effective communication and engagement between researchers and study participants. (4) Value from Participation and Constructive Feedback, capturing the dual focus on the value participants gain from their involvement in the study, and their constructive suggestions. **Conclusions**: This study highlights trust, communication, and emotional impact in neonatal research, emphasising ethical, family-centred design to improve engagement and recruitment in future studies.

## 1. Introduction

Bronchopulmonary dysplasia (BPD) represents one of the most significant complications affecting preterm infants, particularly those born before 32 weeks of gestational age. This chronic lung disease, first described by Northway and colleagues in 1967, continues to pose substantial challenges for neonatal care providers, families, and healthcare systems worldwide [1]. The condition affects approximately one-third of preterm infants born before 32 weeks of gestation in the United Kingdom, translating to roughly seven thousand affected infants annually [2]. The implications of BPD extend far beyond the immediate neonatal period, with affected infants experiencing prolonged hospital stays, increased risk of neurodevelopmental complications, and long-term respiratory morbidities that can persist into adulthood [3].

The pathophysiology of BPD has evolved significantly since its initial description, with contemporary understanding emphasising the vascular theory of disease development. Current evidence suggests that pathological processes affecting the pulmonary vascular bed occur prior to changes in the pulmonary parenchyma, indicating that BPD begins as a vascular disease rather than primarily affecting lung tissue [4]. This understanding has profound implications for early detection and intervention strategies, as it suggests that cardiovascular changes may be detectable before clinical manifestations of respiratory compromise become apparent [5].

The diagnostic criteria for BPD, as established by various consensus statements, rely primarily on oxygen requirements and respiratory support needs at 36 weeks postmenstrual age [6]. However, this approach presents significant limitations, as formal diagnosis cannot be established until this gestational milestone, potentially missing critical windows for early intervention. The delay in diagnosis is particularly problematic given that data from large cohorts demonstrate that the proportion of preterm infants requiring oxygen supplementation decreases from birth to the seventh day of postnatal life, followed by a steep rise beginning in the second week of life, with peak oxygen requirements occurring around the 14th day of postnatal life [7].

### 1.1. The REPORT-BPD Study Context

The Right vEntricular function applicability in a Prediction mOdel to identify pReterm infanTs with early Broncho-Pulmonary Dysplasia (REPORT-BPD) feasibility study represents an innovative approach to addressing the challenges of early BPD detection. This mixed-methods observational cohort study was designed to explore the feasibility of measuring right ventricular function and other echocardiography (echo) parameters in premature hearts to develop a prediction model for identifying early BPD in preterm infants [8]. The study’s foundation rests on emerging evidence that the immature right ventricle of preterm infants may be particularly sensitive to the hemodynamic changes associated with early pulmonary vascular disease, potentially serving as an early marker for BPD development [9].

### 1.2. Family-Centred Care and Research Participation

Contemporary neonatal practice has increasingly embraced family-centred care principles, emphasising parental engagement in infant care, communication, and decision-making processes [10,11,12]. This paradigm shift recognises parents as essential partners in their infant’s care and acknowledges the profound impact that neonatal intensive care unit (NICU) experiences have on families. However, despite this evolution in clinical practice, limited research exists examining the experiences of parents and healthcare professionals involved in neonatal research studies [13].

The importance of understanding stakeholder perspectives in neonatal research cannot be overstated. Parents of critically ill preterm infants face unprecedented stress, uncertainty, and emotional challenges while navigating complex medical decisions for their vulnerable children. When research participation is introduced into this already overwhelming context, it adds additional layers of complexity that require careful consideration and understanding. Healthcare professionals, meanwhile, must balance their roles as caregivers and research facilitators while maintaining therapeutic relationships with families and ensuring optimal patient care.

### 1.3. The Role of Embedded Qualitative Research

The REPORT-BPD study incorporated an embedded qualitative study as a critical component of its feasibility assessment, recognising that quantitative feasibility results alone is insufficient for determining the viability of future large-scale trials. Embedded qualitative studies within feasibility trials serve multiple essential functions: they unveil potential deficiencies prior to full-scale implementation of a larger trial, assess the acceptability of study procedures to participants, and identify barriers and facilitators that may influence recruitment, retention, and data quality in subsequent trials [14].

The qualitative component of the REPORT-BPD study was designed as a two-pronged approach encompassing Work Package II, which examined parental perceptions through NICU diary analysis [15], and Work Package III, which explored experiences through semi-structured interviews with both parents and healthcare professionals. This comprehensive approach acknowledged that research participation occurs within a complex ecosystem involving multiple stakeholders, each with unique perspectives, concerns, and contributions to the research process. Here, we report on the qualitative study with the interviews of parents and healthcare professionals. The aim of this study was to explore the experiences of parents and healthcare professionals in participating in the REPORT-BPD feasibility study.

The decision to include both parents and healthcare professionals as participants in the qualitative study reflects an understanding that successful neonatal research requires buy-in and engagement from all members of the care team. Parents serve as the primary decision-makers regarding their infant’s research participation, while healthcare professionals function as gatekeepers, facilitators, and implementers of research protocols. The experiences and perspectives of both groups are essential for understanding the full spectrum of factors that influence research feasibility and acceptability.

## 2. Materials and Methods

### 2.1. Research Team and Reflexivity

The qualitative research team consisted of the Chief Investigator (CI) Dr. W. S. Muhsen (W.M.), Professor J. M. Latour (J.M.L.) and Ms. Ana Guillot-Lozano (A.G.-L.). W.M. is an experienced consultant neonatologist and affiliated with the University of Plymouth where he is performing a PhD study, which is the REPORT-BPD feasibility study, including quantitative and qualitative research [8]. W.M. had formal training in qualitative research and thematic analysis via the researcher development programme at the University of Plymouth. J.M.L. is a professor of clinical nursing with expertise in qualitative and quantitative research methodologies and an experienced paediatric and neonatal intensive care nurse. A.G.-L. is a student of the Master in Clinical Research programme and an intercalating medical student at the University of Plymouth.

J.M.L. and W.M. designed the qualitative component and developed the consent and interview guide. Semi-structured interviews were carried out by W.M. The qualitative research semi-structured interviews conducted by the treating clinician are intended to leverage the established rapport and trust between the interviewer (clinician) and the interviewees (parents and healthcare staff). This may also facilitate more open and reflective discussions, particularly on sensitive or complex clinical experiences [16]. The clinician’s understanding of the clinical context also enables more informed and meaningful questioning [17]. To mitigate potential concerns around role conflict or social desirability bias, parents were clearly informed that their responses will not affect their children’s clinical care, and that participation is entirely voluntary. Although clinician-led interviews may introduce power dynamic concerns, these were addressed through clear ethical safeguards, including open dialogue and explicitly assuring parents and staff they were free to share both positive and negative views [18]. W.M., J.M.L., and A.G.-L. carried out the data analysis, including coding and the development of subthemes and themes. They also collaborated in drafting this report.

To ensure rigour, all members of the study team reviewed a sample of transcripts and participated in discussions during coding, subthemes and theme development. These measures contributed to the transparency, credibility, and trustworthiness of the findings.

### 2.2. Study Design

This qualitative study used individual semi-structured interviews to explore the perspectives of the REPORT-BPD study participants and healthcare professionals to provide insights to inform the design of a future definitive trial. The consolidated criteria for reporting qualitative research (COREQ) guidelines were followed to conduct and report this study (Appendix A) [19].

### 2.3. Participant Selection

#### 2.3.1. Sampling

Purposive stratified sampling was used to ensure representation of both parents and healthcare professionals. The study aimed to recruit between 10 and 15 participants from each group (parents and healthcare professionals), depending on data saturation. Interviewed clinicians included doctors and nurses involved at different stages of the REPORT-BPD study.

Parents were eligible if their infant had been recruited into the REPORT-BPD study and if they were able to communicate in English. Exclusion criteria included non-English speakers, participants who had withdrawn from the study, and those who had experienced the death of their infant, as recalling the events around their child’s death could lead to unnecessary emotional distress. Healthcare professionals were eligible if they were permanent staff involved in the REPORT-BPD study procedures (e.g., screening, recruitment, data collection). Temporary staff, such as locum staff, were excluded.

Recruitment for the embedded qualitative research commenced once 50% of the target sample size for the REPORT-BPD study had been reached, meaning at least 20 preterm infants had been enrolled.

#### 2.3.2. Method of Approach

For parents, eligibility screening occurred 2–3 days prior to their infant’s second echocardiogram due, i.e., around the end of the first week after birth of their child. A brief face-to-face conversation was initiated by the CI, followed by provision of a participant information sheet (PIS). Interested parents were invited to a follow-up conversation to ask questions, provide written informed consent and to agree a mutually convenient time and date for the semi-structured interview. Semi-structured interviews were scheduled to occur within seven days after consent was given (i.e., between the 10th–17th Day of Postnatal Life (DoPL)).

The NICU staff who took part in the semi-structured interviews met the inclusion criteria, which required involvement in the REPORT-BPD feasibility study through at least one of the study-related activities such as screening, recruitment, or data collection as part of the neonatal research team. Staff who fell under the exclusion criteria—defined as temporary members of staff, such as locum or bank staff—were not invited to participate [8].

Eligible staff were approached by the CI through face-to-face conversation and provided with a PIS. Questions from interested staff were addressed by the CI. After reviewing the PIS and providing written informed consent, semi-structured interviews were scheduled within one month of consent.

#### 2.3.3. Sample Size

A purposive sample of 20–30 participants in total (including 10–15 neonatal unit staff and 10–15 parents) was initially targeted, with the final number determined by data saturation. Data saturation was assessed iteratively throughout the qualitative interview process. As recruitment neared 10 NICU staff members and 10 parents of infants enrolled in the REPORT-BPD feasibility study (Table 1), it became evident that no new themes or insights were emerging. Thematic saturation was considered achieved, as consistent patterns and responses were observed across participants. Consequently, further interviews were not conducted, as additional data collection was unlikely to provide new or relevant information [20,21].

### 2.4. Setting

This study was conducted at the regional tertiary NICU at the University Hospitals Plymouth NHS Trust (UHP).

### 2.5. Data Collection

The semi-structured interviews were conducted between November 2022 and March 2023. All interviews took place in a quiet and private room at the NICU. An interview guide was developed by the study team from existing literature [14]; this method was chosen for its flexibility and depth, allowing participants to elaborate on their experiences while maintaining consistency across interviews [22]. The interview guide comprised four open-ended questions, each focusing on a key domain: participants’ experiences with the REPORT-BPD feasibility study; suggestions for potential improvements; their views on conducting a larger, follow-on study based on this study; and any additional topics they wished to discuss. Hence, all respondents were asked identical questions in the same sequence, but the interviewer probed inductively on key responses. The interviewer collected field notes and a reflective diary during and after each interview to capture non-verbal cues and interviewer insights.

Interviews lasted between 20 and 45 min and were audio-recorded. All recordings were transcribed ad verbatim by a professional transcriber affiliated with the University of Plymouth, working under a confidentiality agreement. The use of a professional transcriber is supported in qualitative research to enhance transcription accuracy and reduce potential researcher bias [23]. No repeat interviews were conducted.

For parent interviews, both mothers and fathers were invited to participate individually to accommodate caregiving responsibilities. Efforts were made to ensure a balanced representation of maternal and paternal perspectives, recognising the known underrepresentation of fathers in neonatal research [24]. Similarly, equal attention was given to achieving a balanced number of healthcare professionals from both nursing and medical backgrounds to capture a range of professional viewpoints.

#### Member Checking

To enhance credibility, member checking was employed at transcript level [25]. Member checking was conducted with two parents and two healthcare professionals, who were provided with hard copies of their interview transcripts within 1–2 weeks of their interviews. They were invited to review and clarify any part of the content. Participant validation process was considered convenient, as parents were either resident or frequent visitors to the NICU, and staff members held permanent or fixed-term positions within the department. Broader member checking of data analysis was not undertaken due to practical and ethical considerations, including the potential emotional burden on parents and the risk of participant attrition [25].

### 2.6. Data Analysis

Thematic analysis followed the six-step framework outlined by Braun and Clarke (2006) and was conducted by using NVivo software (Version 14; Lumivero, Denver, CO, USA) to support data organisation. Initial codes were developed inductively from the transcripts [26]. To ensure the credibility and dependability of the analysis, two members of the research team independently reviewed a sample of the transcripts and participated in discussions about coding, subthemes, theme development, and refinement. This collaborative approach helped challenge assumptions, confirm interpretations, and strengthen analytical rigour.

## 3. Results

Four themes were developed from the interview data, reflecting the experiences and perceptions of parents and healthcare professionals who participated in the REPORT-BPD feasibility study. Each theme is described alongside its subthemes and supported by direct participant quotations to ensure transparency and consistency with the data (Table 2).

### 3.1. Themes and Subthemes

#### 3.1.1. Trust and Assurance in the Study

This theme describes the confidence participants expressed in the ethical integrity, safety, and low burden of the study, which fostered their willingness to participate and trust in the research process.

##### Confidence in the Study’s Safety and Benefits

Participants believed the study was conducted safely and viewed it as beneficial to both their child and the broader neonatal population.

“*As a parent, I am fine with it and happy with the way it turned out, and yeah, all the additional bits that came with it, I think will be good for him and good for the study as well*”

(Parent 003)

“*It is absolutely worthwhile, because the neonatal population, if we can prevent the… the big sequelae of extremely preterm birth, and if we can prevent that by surveillance like echocardiography and a management plan, the ongoing care for these preterm babies will; A—support them and their parents to have a less than medicalised lifestyle.*”

(HCP 007)

The parents’ remarks demonstrated a thoughtful appreciation of the importance of neonatal research, given the persistent challenge of prematurity. Their comments highlighted that robust, well-designed research is a critical factor in enhancing the quality of neonatal care and generating favourable outcomes. This was further supported by strong endorsement by healthcare professionals, which played a crucial role in enabling the successful completion of participants’ recruitment ahead of the scheduled deadline.

##### Acknowledging the Study Did Not Add Extra Burden on the Infant

Many parents felt that their child’s routine care was not disrupted by the study, which aligned with the staff’s view.

“*it’s been part of the broader care, so it’s not like it’s been an obvious hinderance*”

(Parent 007)

“*The one good thing about the study is the fact that it’s actually minimalistic in the sense that we’re not having to take blood tests and it’s not one of those where you’re having to take loads and loads of samples from… from the patient.*”

(HCP 009)

Communication with parents revealed that a primary concern was uncertainty about potential harm to their preterm infants associated with research participation (Table 2). During the recruitment process, researchers addressed these concerns through structured counselling, emphasising the rigorous safeguards in place to ensure participant safety. This reassurance was reinforced when parents observed that research involvement did not impose additional burdens on their child, particularly as study procedures such as heart scans were integrated into routine clinical care and carried both clinical and research value.

##### The Consenting Process Was Administered with Satisfactory Rigour

Parents and staff praised the thoroughness and professionalism of the consent process.

“*You made it very clear what is going to happen and that first piece of paper we the points on it were very clear as well*”

(Parent 005)

“*I’d be happy to kind of provide them with information as well that they could take away so I know we have the information leaflets so I could go with that in hand and that could help prompt me to talking to them about it as well. So, yeah, if I used the right things to aid me, then yeah, definitely.*”

(HCP 003)

Active engagement of both parents and healthcare professionals was evident in the informed consent process, including adherence to the study timeline and prompt efforts to comprehend and finalise consent documentation within the initial days after birth.

#### 3.1.2. Communication and Engagement

This theme underscores the importance of effective communication and engagement between researchers and participants, addressing knowledge gaps and fostering collaboration for future studies.

##### Ensuring Clear and Meaningful Communication with Parents

Effective explanations and clarity contributed to a sense of informed participation, although some variability was noted.

“*It has been fantastic, you kept us in the loop about everything that we need to know and this is what we can have really, and you took the correct step*”

(Parent 002)

“*I think the research team have been really, I guess, in depth with their explanation in terms of what the study aims to achieve, how the study is conducted, how the infants will… you know, like what will happen with the infants in the study as part as that, and answering parents’ questions and things like that.*”

(HCP 005)

##### Exploring Participants Expressed Knowledge Gaps and Limited Understanding

Despite receiving information, some participants reported limited understanding of the study’s purpose or procedures.

“*But I… I guess ultimately to try and find a way to be able to pick up potential lung issues just through the heart rate, and at earlier stages, so you can pick it up at an earlier stage. That’s… that’s… that’s my understanding of it, from what you’ve explained to me, yeah.*”

(Parent 007)

“*I know it’s about monitoring their PDAs and all that, but obviously I don’t have like, the main purpose, what will help them, what will be the future help for their babies. So, I’m not really fully equipped to explain it to the parents, so…*”

(HCP 008)

Interestingly, the interviews revealed a disparity in understanding the study between parents and healthcare professionals. While parents demonstrated a good grasp of the research objectives, healthcare professionals exhibited variable levels of awareness. This difference may be attributed to the timing of data collection: parents were interviewed shortly after completion of the second heart scan, when study details were still salient, whereas interviews with healthcare professionals were conducted several weeks later. Additionally, parents received regular updates from the clinical team, including information on study-related procedures such as cardiac imaging, while no equivalent system of ongoing communication was in place for staff, limiting opportunities to reinforce study knowledge.

##### Parents and Staff Express Willingness to Support a Future Large-Scale Study

Many participants expressed support for scaling up the study, viewing it as worthwhile and important.

“*If it helps a little bit to progress something in the future, I’d love to be part of that trying to push that in the future, more than happy yeah.*”

(Parent 002)

“*I’d definitely be keen to get involved if it was rolled out.*”

(HCP 001)

These findings indicate a favourable disposition among both parents and healthcare professionals toward involvement in neonatal research, including support for large-scale clinical trials designed to expand evidence-based practices in neonatal care.

The Communication and Engagement theme provides crucial insights into the complex dynamics of information sharing and understanding in neonatal research contexts (2). The finding that effective communication fostered informed participation while knowledge gaps persisted despite information provision highlights the ongoing challenge of ensuring true informed consent in complex clinical research. The identification of specific knowledge gaps provides actionable targets for improvement in future studies, including enhanced PIS and more targeted educational interventions, e.g., recorded presentation regarding the study information.

#### 3.1.3. Emotional and Psychological Considerations

This theme reflects the emotional challenges and coping mechanisms described by parents navigating the neonatal environment, and how these shaped their views on research participation.

##### Concerned About Their Child’s Future Well-Being

The emotional strain of having a vulnerable infant influenced parents’ decision-making processes about the study.

“*Umm, do you know it went really well from the start to finish and I feel that he had a good care from the get-go, at times, I was really worried about taking the next step but everyone was reassuring, and we couldn’t complain with the care so far so yeah, really positive.*”

(Parent 001)

It is essential to carefully balance communication with parents, taking into account the significant emotional stress they experience. Providing parents with sufficient time to process information, along with the space to adapt to their new reality, is crucial—supported consistently by the neonatal care team.

##### Factors That Alleviate Parental Stress

Supportive interactions and research staff reassurance played a role in reducing stress.

“*Yeah, yeah, about the diary and stuff. So, it’s wonderful, it’s lovely, it’s… it’s actually quite therapeutic in helping to sort of sit there and actually… it… it’s that sort of offloading to a degree, which is a great thing in this environment anyway when there’s so much going on.*”

(Parent 007)

One key finding that emerged from the interviews was the perceived therapeutic value of the study intervention. Parents described the neonatal research diary as a reflective tool and a dedicated opportunity to sit down, document their experiences, and emotionally offload, thereby supporting psychological coping during their infant’s hospitalisation.

##### The Challenges Encountered by Parents in the Neonatal Unit

Parents shared accounts of the emotional and logistical hardships associated with participating on the study during their NICU stays.

“*It was just the shock of the situation that we were in as we didn’t expect to have 25 weeks baby and life become like a whirlwind and you become almost lost in yourself, if that makes sense.*”

(Parent 005)

“*But I wasn’t in the right sort of place to write down my emotions.*”

(Parent 005)

“*Well, my half-asleep brain isn’t processing.*”

(Parent 009)

This further underlines the considerable emotional burden experienced by parents and highlights the need for structured support mechanisms, providing valuable guidance for the neonatal team in delivering empathetic and family-centred care.

#### 3.1.4. Value from Participation and Constructive Feedback

This theme captures the dual focus on the value participants gain from their involvement in the study, as well as their constructive suggestions. It highlights how participant insights can enhance future research while recognising the value of their involvement.

##### Perceived Advantages from Participating in the Study

Participants found personal and altruistic value in their involvement.

“*Parents quite often like having the extra information about their baby, and if it then leads to something that will then give us a better idea of where we’re going in terms of prediction, I think it’s useful.*”

(HCP 004)

##### Potentially Beneficial Suggestions for Improvements

Participants offered actionable feedback on aspects like study information clarity and timing of approach.

“*Umm…So, maybe for the next notebook, just add the odd questions there just to prompt the parents if they are unsure of what to put in there, I suppose.*

*I don’t know if this will be the right thing for you guys? But it seems I managed it better than my partner did, I would say.*”

(Parent 006)

Parents and healthcare professionals provided valuable insights into both the benefits of the study and potential areas for improvement. Healthcare professionals noted that parents appreciated ongoing updates regarding their infant’s condition, including clear explanations of clinically indicated procedures such as cardiac imaging. These communications were perceived to enhance parental understanding and engagement.

Furthermore, healthcare professionals noted that the study provided a valuable opportunity for medical and nursing trainees to gain observational exposure to neonatal research, including witnessing key processes such as patient recruitment and study procedures.

Parents also contributed constructive suggestions for refining the research process. For instance, they recommended incorporating open-ended guiding questions into the neonatal research diary to support reflective writing. Such a modification could enhance the diary’s utility as a therapeutic and research tool, while reducing the cognitive burden on participants.

## 4. Discussion

This embedded qualitative study generates several important contributions to our understanding of stakeholder experiences in neonatal research and provides actionable insights for future trial design. The four overarching themes that emerged from the analysis—Trust and Assurance in the Study, Communication and Engagement, Emotional and Psychological Considerations, and Value from Participation and Constructive Feedback—collectively paint a comprehensive picture of the factors that influence participation in neonatal feasibility studies and provide a roadmap for optimising future research endeavours.

Interview findings highlighted the importance of fostering trust between parents and healthcare professionals throughout the research process. Open and transparent communication was central to establishing and maintaining this trust, beginning at the initial stages of recruitment and continuing through to the completion of study procedures. This aligns with prior , that trust is a necessity for ethically sound consent in neonatal research, where parents face particularly vulnerable decision-making contexts [27]. Endorsement of the study by healthcare professionals, along with their willingness to support its implementation, played a key role in facilitating successful study completion [28]. Furthermore, regular communications and ongoing reassurance effectively identified and addressed parental concerns—such as potential study burdens—and contributed to enhancing their sense of responsibility, reflected in their active, collaborative engagement in the consent process and the research overall [28]. These findings support existing evidence that continuous, transparent engagement sustains trust and encourages participation [29,30].

Interestingly, parents had a fair understanding of the study, whereas awareness among healthcare professionals varied considerably. This disparity may be attributed to differences in the timing of interviews relative to study engagement. Although all interviews were conducted during the active recruitment phase, parental interviews took place shortly after the second cardiac scan—when study procedures and outcomes were still salient. In contrast, interviews with staff were conducted several weeks after their involvement in recruitment. Additionally, parents received regular updates through clinical feedback, including results from the heart scans, which reinforced their understanding. In comparison, there was a lack of ongoing communication regarding the REPORT-BPD feasibility study following the initial training and presentation sessions, likely contributing to knowledge gaps among the clinical team. Hence, regular dissemination of research updates to all stakeholders, such as regular refresher sessions for healthcare professionals, digital bulletins, or embedded research champions, is essential to sustain focus, ensure alignment with study objectives, and maintain the relevance and rigour of data collection.

The findings further revealed that reflective journalling in the neonatal research diary was experienced as a therapeutic activity, supporting emotional processing and fostering resilience during a period of significant stress.

The perspectives of both parents and healthcare professionals yielded important insights into the study’s impact and opportunities for refinement. A key facilitator of parental engagement was the provision of timely and transparent updates regarding their infant’s clinical course, including clear explanations of procedures such as cardiac imaging. These communications were consistently valued by parents and perceived by staff as instrumental in fostering trust, enhancing understanding, and supporting informed participation—findings that echo wider calls for feedback loops in neonatal research [29].

Beyond direct participant engagement, the study served as an educational platform for medical and nursing trainees, offering observational exposure to the conduct of neonatal research. Witnessing processes such as patient recruitment and protocol implementation provided trainees with practical insight into research ethics, communication strategies, and interdisciplinary collaboration—experiences that are often underrepresented in clinical training.

Parents also offered meaningful suggestions for improving study procedures. Notably, several recommended the inclusion of open-ended, guiding questions in the neonatal research diary to support reflective writing. This feedback highlights the cognitive and emotional challenges parents may face when journalling during periods of high stress. Incorporating structured prompts could not only improve data quality but also enhance the diary’s dual role as both a research instrument and a supportive tool for emotional processing and their therapeutic potential of expressive writing in high-acuity care settings.

### 4.1. Study Limitations

While this embedded qualitative study provides valuable insights into stakeholder experiences with the REPORT-BPD feasibility study, several limitations must be acknowledged that may affect the interpretation and generalizability of the findings.

First, the single-site design limits the generalizability of findings to other neonatal intensive care settings. The study was conducted exclusively at a regional tertiary NICU, which may have unique characteristics in terms of staffing patterns, clinical protocols, organisational culture, and patient populations that could influence participant experiences. Future research would benefit from multi-site studies that can capture variation in experiences across different organisational and cultural contexts [31].

Second, the exclusion of non-English speaking participants may have resulted in the omission of important perspectives. This exclusion criterion, while practically necessary given resource constraints, may have systematically excluded participants from certain ethnic and cultural backgrounds who might have different experiences with research participation, different communication preferences, or different cultural perspectives on medical decision-making for their preterm infants. The absence of these perspectives limits the comprehensiveness of the findings and may result in recommendations that are less applicable to diverse populations. Future studies should consider incorporating translation services and culturally appropriate research methods to ensure more inclusive participation [32,33].

Third, due to limited resources, all interviews were conducted by a single interviewer. While having one consistent interviewer ensured standardisation of the interview process and maintained continuity across all participant interactions, this approach may have limited the range of perspectives and questioning styles that could have enriched the data collection process (6). Different interviewers might have pursued different lines of inquiry, asked follow-up questions from varying perspectives, or established different rapport dynamics with participants, potentially uncovering additional insights or themes that were not captured by a single interviewer’s approach. Future studies would benefit from employing multiple trained interviewers to enhance the robustness of data collection and reduce the potential for interviewer-related bias.

### 4.2. Study Strengths

Our study demonstrated several methodological strengths that enhance credibility, trustworthiness, and utility of its findings for informing future research endeavours.

The study’s rigorous methodological approach, following established qualitative research guidelines and best practices, represents a significant strength that enhances the credibility of the findings. The research team adhered to the Consolidated Criteria for Reporting Qualitative Research (COREQ) guidelines, ensuring comprehensive and transparent reporting of all aspects of the research process (Appendix A) [17,19]. The use of Braun and Clarke’s well-established six-step framework for thematic analysis provided a systematic and rigorous approach to data analysis that enhances the dependability of the findings. The involvement of multiple researchers in the coding and theme development process, with independent review of transcripts and collaborative discussions about interpretation, strengthens the analytical rigour and reduces the potential for individual researcher bias [17]. The implementation of member checking at the transcript level with selected participants further enhanced the credibility of the data by allowing participants to verify the accuracy of their recorded responses [25].

The achievement of data saturation with an appropriate sample size demonstrates the study’s methodological soundness and suggests that the findings comprehensively capture the range of relevant experiences and perspectives [20,21]. The purposive stratified sampling approach ensured balanced representation of both key groups (parents and healthcare professionals) and achieved diversity within each group, including balanced representation of maternal and paternal perspectives and equal representation of nursing and medical professionals.

Unlike standalone qualitative studies that may provide general insights into stakeholder perspectives, this embedded approach captured experiences directly related to specific study procedures and contexts, making the findings immediately applicable to future trial optimisation. The timing of interviews during the active phase of the feasibility study allowed for real-time capture of experiences while they were still fresh and relevant, avoiding the potential for recall bias or post hoc rationalisation that might affect retrospective qualitative research [17].

## 5. Conclusions

The study findings collectively generate several important insights for the broader field of neonatal research. First, they demonstrate that complex research procedures can be successfully integrated into routine neonatal care when designed thoughtfully and implemented with appropriate stakeholder engagement. Second, they highlight the critical importance of communication and relationship-building in establishing trust. Third, they provide specific guidance for addressing the unique emotional and practical challenges associated with research participation in the NICU environment.

## Figures and Tables

**Table 1:** Characteristics of parents and neonatal staff and parents.

Participant	Staff or Family	Sex
QP-001	Parent	F
QP-002	Parent	M
QP-003	Parent	M
QP-004	Parent	F
QP-005	Parent	M
QP-006	Parent	F
QP-007	Parent	M
QP-008	Parent	F
QP-009	Parent	F
QP-010	Parent	M
QS-001	Staff/Medical background	M
QS-002	Staff/Medical background	F
QS-003	Staff/Nursing background	F
QS-004	Staff/Medical background	F
QS-005	Staff/Nursing background	F
QS-006	Staff/Medical background	F
QS-007	Staff/Nursing background	F
QS-008	Staff/Nursing background	F
QS-009	Staff/Medical background	M
QS-010	Staff/Nursing background	F

F = Female; M = Male; QS = Qualitative research Staff participant QP = Qualitative research Parent participant.

**Table 2:** Themes and subthemes with the related quotations.

Themes	Subthemes	Quotations
**Theme 1**: Trust and Assurance in the Study	Confidence in the study’s safety and benefits.	*“We were quite happy to get into the study just purely from the fact that obviously she’ll be getting a little bit more insight as to how her body is developing, and I think that’s always a good thing, you know”—Parent 010* *“I think, you know, if we can identify those babies that are more likely to develop BPD and as a result potentially look at ways of intervening earlier to, you know, minimise the extent of it, I think that’s got to be good for babies.”—HCP 001*
	Acknowledging the study did not add extra burden on the infant.	*“So, it is only positive in my opinion and more people you ask and more people you see get to do this sort of thing will only get better results, yeah.”—Parent 001* *“it’s not a study that’s any harm really to the baby.”—HCP 004*
	The consenting process was administered with satisfactory rigour.	*“We didn’t feel pushed into it or forced or had nothing in that sort of lines.”—Parent 006* *“the good part is that baby is there and we need to get the consent before day five, so usually mum is there as well, and usually the… a partner is there, so it’s quite easy to get hold of them, and if… it’s sometimes difficult overnight because they’re sleeping or unwell and do not want to talk, especially on day one of life, you still have the other three days, and you definitely can catch up with them.”—HCP 002*
**Theme 2**: Communication and Engagement	Ensuring clear and meaningful communication with parents.	*“Umm… so you mentioned, you know, that we could pop in later on and either chat to you or be there when the scans are happening, that is always reassuring.”—Parent 006* *“Yeah, it’s all explained, it’s quite easy to understand, and then obviously if they’ve got any questions, they could come to you, and you will clarify anything that they need.”—HCP 010*
	Exploring participants expressed knowledge gaps and their limited understanding of the study’s aim and procedures.	*“You know, but I’m not 100% certain. I haven’t done any research myself into what, you know, the study mainly focused on.”—Parent 010* *“But before I give parents anything, I always read it again myself to refresh myself on everything before I present it to the parents.”—HCP 006*
	Parents and staff express willingness to support a future large-scale study.	*“Even if it didn’t get to a larger study, even the smaller scale study can teach doctors, parents and everyone. I think larger scale study can definitely improve upon that. And with the right elements, equipment and everything else that could really benefit babies and future generations as well. I would say, it will definitely be a good idea”—Parent 003* *“Well, it depends on what findings you have of the smaller ones, isn’t it, and if that… if that doesn’t really help much then maybe a bigger study will be helpful, and that’s… yeah, yeah.”—HCP 008*
**Theme 3**: Emotional and Psychological Considerations	Concerned about their child’s future well-being.	*“Some of the wording, while was fine and understood, it gave a little bit of a worry to me and BABY’s mum when it said that some can go wrong and he could be harmed in any way”—Parent 003*
	Factors that alleviate parental stress.	*“I honestly don’t mind, because you all know what you’re talking about, so that’s… as long as I heard about the information at some point, because it’s always… always reassuring to hear, it was fine no matter who it came from really, yeah.”—Parent 008*
	The challenges encountered by parents in the neonatal unit.	*“Which makes it hard to remember. But then I… I have all the written things, if I wanted to look back.”—Parent 009* *“You probably did explain it all, but because I didn’t have any sleep for a few nights…”—Parent 009*
**Theme 4**: Value from Participation and Constructive Feedback	Perceived advantages from participating in the study.	*“The trainees need to… yeah, definitely be fully informed about this, because I keep telling them it’s a good thing for them to be involved with, because you know, also it’s part of research, you’re taking part in a research project, aren’t you. So, whilst you haven’t designed it yourself, you have helped to actively recruit some patients for it, so…”—HCP 006*
	Potentially beneficial suggestions for improvements.	*“Yeah, the only thing that might be changed is regarding the neonatal unit/research diary. I guess that the research team wanted to leave it as open-ended one/blank, which makes a lot of sense. But I found it really difficult to write in it, while my partner could easily write in it. I guess I found it hard to know what I need to write that sort of thing.”—Parent 005*

## Data Availability

The raw data supporting the conclusions of this article will be made available by the authors on request.

## Abbreviations

The following abbreviations are used in this manuscript:

BPD	Bronchopulmonary Dysplasia
CI	Chief Investigator
COREQ	Consolidated Criteria for Reporting Qualitative Research
HCP	Healthcare Professional
NICU	Neonatal Intensive Care Unit
NVivo	(Not an abbreviation: Qualitative Data Analysis Software)
PIS	Participant Information Sheet
REPORT-BPD	Right vEntricular function applicability in a Prediction mOdel to identify pReterm infanTs with early BronchoPulmonary Dysplasia
UHP	University Hospitals Plymouth NHS Trust
WP-II	Work Package II
WP-III	Work Package III

## Appendix A. COREQ Checklist

**Domain**	**Item #**	**COREQ Item**	**Response for This Study**
**1. Research Team and Reflexivity**	1	Interviewer/facilitator: Which author(s)/researcher(s) conducted the interview or focus group?	Dr. Wisam Muhsen (WM), the Chief Investigator, conducted all semi-structured interviews. WM is a consultant neonatologist and PhD candidate with formal training in qualitative research.
	2	Credentials: “What were the researcher’s credentials (e.g., academic position, discipline, qualifications)?”	WM: Consultant neonatologist, PhD candidate in clinical research (University of Plymouth). Prof. Jos M. Latour (JML): Professor of Clinical Nursing, experienced in qualitative and quantitative methodologies. Ms. Ana Guillot-Lozano (AGL): Intercalating medical student and Master’s student in Clinical Research.
	3	Occupation: What was their occupation at the time of the study?	WM: Clinician-researcher (neonatologist) and PhD student. JML: Academic professor and researcher. AGL: Medical student and master’s degree student.
	4	Gender: Was the interviewer male or female?	Male.
	5	Experience and training: What experience or training did the interviewer have?	WM received formal training in qualitative methods and thematic analysis through the University of Plymouth’s Researcher Development Programme. He also has extensive clinical experience in neonatal care.
	6	Relationship established	The relationship was established prior to the study commencement
	7	Participants knowledge of the interviewer?	WM was the treating clinician for the infants whose parents were interviewed. Staff participants were colleagues within the same NICU. This dual role was acknowledged, and ethical safeguards were implemented (e.g., voluntary participation, assurance that responses would not affect clinical care).
	8	Interviewer characteristics	The interviewer is an experienced consultant neonatologist and clinical researcher who is interested in the investigating Bronchopulmonary dysplasia affecting newborn infants.
**2. Study Design**	9	Methodological orientation and theory?	Qualitative descriptive design using thematic analysis following Braun & Clarke’s (2006) [26] six-step framework.
	10	Sampling: How were participants sampled?	Purposive stratified sampling to ensure diversity in gender (parents) and professional background (medical vs. nursing staff).
	11	Method of approach?	Participants were approached face-to-face.
	12	Sample size: How many participants were in the study?	20 participants: 10 parents and 10 healthcare professionals.
	13	Non-participation: Were any participants not willing or able to participate?	Non-English speakers and families who experienced infant death were excluded. All approached eligible participants consented.
	14	Setting of data collection.	Semi-structured individual interviews. All interviews were conducted in the neonatal unit private room.
	15	Presence of non-participants.	Only the researcher and the participants were present in the interviews.
	16	Description of sample	Demographic data were collected e.g., gender, parent, medical or nursing background.
	17	Interview guide	There was an interview guide established with the questions included.
	18	Repeat interviews	There was no repeat interview.
	19	Audio/visual recording.	All interviews were audio-recorded.
	20	Field notes: Were field notes made during or after interviews?	Yes. The interviewer maintained field notes during and after each interview to capture non-verbal cues and contextual insights.
	21	Duration: What was the duration of interviews?	Interviews lasted 20–45 min.
	22	Data saturation: Was data saturation discussed?	Yes. Saturation was assessed iteratively.
	23	Transcripts returned: Were transcripts returned to participants for comment or correction?	Transcript-level member checking was conducted with 2 parents and 2 staff.
**3. Analysis and Findings**	24	Number of data coders: How many researchers coded the data?	Three researchers (WM, JML, AGL) collaboratively coded the data and developed themes.
	25	Description of the coding tree: Was a coding tree or framework described?	Initial codes were developed inductively. Subthemes and themes were refined through team discussion. The final thematic structure (4 themes, 11 subthemes) is presented in Table 2.
	26	Derivation of themes: Were themes derived inductively or deductively?	Inductively, from the raw data.
	27	Software: Was software used for data management or analysis?	NVivo 14 was used to manage and support thematic analysis.
	28	Participant checking	Transcript-level member checking was conducted with 2 parents and 2 staff. But no participant checking regarding the findings was performed.
	29	Participants’ quotes: Are participant quotations presented to illustrate themes?	Yes. Direct quotations from both parents and healthcare professionals are provided for each subtheme in Table 2 and throughout the Results section.
	30	Data and findings consistency: Do quotes match the findings?	Yes. Quotes are contextually aligned with the themes and subthemes they represent.
	31	Clarity of major themes.	Yes. Four main themes are clearly articulated and well-supported with relevant participant quotations and contextual interpretation.
	32	Clarity of minor or subthemes.	Yes. 11 subthemes are clearly described and illustrated through direct quotes and explanatory narrative.
